# The Role of GLI in the Regulation of Hepatic Epithelial–Mesenchymal Transition in Biliary Atresia

**DOI:** 10.3389/fped.2022.861826

**Published:** 2022-05-26

**Authors:** Pu Siyu, Wang Junxiang, Wang Qi, Zhang Yimao, Jin Shuguang

**Affiliations:** Department of Pediatric Surgery, West China Hospital of Sichuan University, Chengdu, China

**Keywords:** biliary atresia, epithelial–mesenchymal transition, Shh signaling pathway, GLI, liver fibrosis

## Abstract

**Objective:**

To study the regulatory role of GLI1/GLI2, a nuclear transcription factor of the Sonic hedgehog (Shh) signaling pathway, in epithelial–mesenchymal transition (EMT) related to hepatic fibrosis in patients with biliary atresia (BA).

**Methods:**

The messenger RNA (mRNA) and protein expression levels of GLI1/GLI2, Snail/Slug, and other Shh- and EMT-related cytokines were tested in the liver tissues of BA patients and animals. Then, GLI1/GLI2 was silenced and overexpressed in mouse intrahepatic bile duct epithelial cells (mIBECs) and BA animals to investigate changes in the mRNA and protein expression of EMT key factors and liver fibrosis indicators. After silencing and overexpression of GLI1/GLI2, immunofluorescence was used to detect the expression of cytokeratin-19 (CK19) and α-smooth muscle actin (α-SMA) in mIBECs, and hematoxylin and eosin (HE) staining and Masson staining were used to observe the degree of liver fibrosis in the BA animals.

**Results:**

Compared with the control, the mRNA and protein expression levels of GLI2, Snail, vimentin, and α-SMA were significantly increased and those of E-cadherin were significantly decreased in liver tissue from BA patients and animals. Overexpression of GLI2 increased the mRNA and protein expression levels of Snail, vimentin, and α-SMA and that of E-cadherin was significantly decreased in mIBECs and BA animals. After GLI2 silencing, the opposite pattern was observed. Immunofluorescence detection showed enhanced expression of the bile duct epithelial cell marker CK19 in mIBECs after GLI2 silencing and enhanced expression of the mesenchymal cell marker α-SMA after GLI2 overexpression. HE and Masson staining suggested that the GLI2-overexpressing group had a significantly higher degree of fibrosis.

**Conclusion:**

The Shh signaling pathway plays an important role in fibrogenesis in BA. GLI2 can significantly regulate EMT in mIBECs and livers of BA mice.

## Introduction

Biliary atresia (BA) is the most common cause of neonatal cholestasis, characterized by persistent inflammation of the intra- and extrahepatic bile ducts with rapid progression to liver fibrosis (1). Children who do not receive treatment usually die before the age of 2 years due to cholestasis and progressive liver fibrosis (2). The etiology of BA is multifactorial, with a number of possible pathomechanisms being proposed, including viral infections, toxins, genetic susceptibility, immunogenic abnormalities, maternal microchimerism, vascular disturbances, and abnormal remodeling processes (3–5). However, the most accepted hypothesis at present is that BA is a virus-induced autoimmune disease (1, 6).

The inflammatory fibrosis process in bile ducts is regulated by several pathways, such as the Notch and transforming growth factor-β/Smad signaling pathways (7). Recent studies have shown that the Sonic hedgehog (Shh) signaling pathway and epithelial–mesenchymal transition (EMT) may also play an important role in this process (8, 9). The Shh signaling pathway is involved in embryonic development, and several studies have suggested that the GLI family, the crucial factors of the Shh pathway, is highly expressed in the BA process (10, 11). EMT is the process of epithelial polarized cell dedifferentiation and conversion to motile mesenchymal-appearing cells, during which a series of changes occur, including loss of intercellular contacts and cell polarity, as well as alterations of various molecular markers, including E-cadherin, vimentin, α–smooth muscle actin (α-SMA), cytokeratin-19 (CK19), etc. (12, 13). The Snail family of zinc-finger transcription factors plays an evolutionarily conserved role in vertebrate mesoderm formation, of which Snail 1 (Snail) and Snail 2 (Slug) act as key inducers of EMT (14, 15).

The present study was designed to verify the existence of Shh and EMT in hepatic fibrosis of BA by detecting the expression of Shh- and EMT-related factors in BA liver biopsy samples and to investigate the relationship between the two and the specific regulatory mode of regulatory factors involved in the fibrosis process to find new ideas for the prevention and treatment of liver fibrosis in BA.

## Materials and Methods

### Reagents and Antibodies

Primers were synthesized by Sangon Biotech Co. (Shanghai, China). Antibodies against GLI1, GLI2, Snail, Slug, E-cadherin (*CDH1*), vimentin, α-SMA, CK19, and secondary antibodies were obtained from Cell Signaling Technology, Inc. (Beverly, MA, United States). BALB/c mice, 5 weeks old, were purchased from Dossy Experimental Animals Co. (Chengdu, China). HEK293 cells (human embryo kidney cells) were obtained from the American ATCC Cell Line Center.

### Liver Tissue Collection and Patient Characteristics

Ten liver biopsies were obtained from BA patients with surgically confirmed type III between January and December 2018 and were selected for this study. The patients (four males and six females) were aged 1–3 months. Liver tissue samples obtained during autopsy from five children without hepatic and biliary malformation in the same age group were included in analyses as normal controls. All subjects were ethnic Chinese. This study was performed according to a protocol that conformed to the ethical guidelines of the 1975 Declaration of Helsinki Declaration of the World Medical Association and approved by the Institutional Review Board and Ethical Committee at the West China Hospital of Sichuan University in China. Written informed consent was obtained from the parents prior to specimen collection.

The collected liver samples were divided into two parts: one part was fixed overnight in 10% neutral buffer formalin, followed by paraffin embedding for immunofluorescence and histology, and the other part was rapidly frozen in liquid nitrogen and stored until analysis.

### Generation of Recombinant Viruses

Adenoviruses-Gli1/Gli2 (ADV-Gli1/2): *Gli1* and *Gli2* cDNAs of mice were inserted into adenovirus vector PAD-track-CMV (Beyotime, Shanghai, China) and cotransfected with shuttle plasmid AD-Easy1 (Beyotime, Shanghai, China) into recombinant BJ5183 (Beyotime, Shanghai, China) to obtain the positive clones. After amplification, high purity endotoxin-free extraction was performed, followed by transfection of 293T cells (Sigma, Shanghai, China). After 48 h of culture, virus-rich cells were collected from the supernatant by centrifugation and stored at −80°C until thawing for subsequent experiments. Following three cycles of freezing/thawing, 5 μl of viral lysate was used to detect the *Gli1* and *Gli2* genes in adenoviral particles with reverse transcription-PCR, and the titer was determined.

Lentivirus-Gli1/Gli2 (Gli1/Gli2-shRNA): a lentiviral shRNA vector targeting *Gli1* and *Gli2* was generated by inserting stranded oligonucleotides (Gli1-shRNA, forward sequence: 5′-GATCCGCAGCATGAGCTCTGCTTACATTCAAGAGATGTA AGCAGAGCTCATGCTGCTTTTTTA-3′ and Gli1-shRNA, forward sequence: 5′-GATCCGTGTGGAGGACTGCCTACATA TTTCAAGAGAATATGTAGGCAGTCCTCCACATTTTTTA-3′) into the lentiviral vector pLent-U6-Puro (Beyotime, Shanghai, China). The other steps were the same as those for ADV-Gli1/2. Lentiviral vectors were diluted into three gradients (10, 1, and 0.1 μl per 500 μl) and used to infect HEK293 cells. Cells were collected 64–68 h postinfection, genomic DNA was extracted, and a quantitative real-time PCR (qPCR) assay was performed to calculate viral titers until vector titers exceeding 10 (8) transduction units/ml could be harvested from the final producer clones.

### Cell Isolation, Culture, and Treatments

Normal mice were anesthetized and cannulated in the portal vein and then perfused with serum-free Dulbecco’s modified Eagle’s medium/F12 (Thermo Fisher Scientific, Waltham, MA, United States) and 2 mg/ml type II collagenase (Sigma, Shanghai, China) to remove blood and most of the hepatocytes. Then, the intrahepatic bile ducts of mice were removed and minced, type IV collagenase (Sigma, Shanghai, China) was added and shaken for 60 min at 37°C, filtered through 200-mesh cell strainers and centrifuged, and the isolated cells were cultured in Dulbecco’s modified Eagle’s medium/F12 medium containing 10% FBS in an incubator at 37°C and 5% CO2. Immunofluorescence of isolated BECs with CK19 to control purity >90% (Supplementary Figure 1).

Cells were infected with ADV-Gli1, ADV-Gli2, Gli1-shRNA, and Gli2-shRNA using Lipo 8000 (Beyotime, Shanghai, China). After validation by western blotting, the cells were divided into seven groups: normal control, adenovirus control, lentivirus control, ADV-Gli1 group (MOI of 50), ADV-Gli2 group (MOI of 50), Gli1-shRNA group (MOI of 40), and Gli2-shRNA group (MOI of 40).

### Animal Preparation and Biliary Atresia Induction

All animal procedures were reviewed and approved by the Laboratory Animal Ethics Committee of the West China Hospital of Sichuan University. Thirty-two SPF-grade BALB/c mice were housed in specific pathogen-free static cages with a 12-h light/dark cycle, with chow pellets and tap water available *ad libitum*. Mice were caged in a 3:1 male to female ratio. The postnatal mice were randomly divided into two groups, and the BA group was injected intraperitoneally with 100 μl of rotavirus fluid (Genechem, Shanghai, China; virus TCID50: TCID50 = 10−7.64/0.2 ml) and the control group with an equal amount of virus maintenance fluid as previously described (16). Neonatal BALB/c mice infected with rotavirus presented with hyperbilirubinemia, acholic stools, inflammation, and obstruction of the extrahepatic bile ducts at 2 weeks of age and were defined as BA mice (17).

The mice at 2 weeks of age were randomly divided into six groups: normal control group, BA model control group, ADV-Gli1 group, ADV-Gli2 group, Gli1-shRNA group, and Gli2-shRNA group. The ADV-Gli1/Gli2 group and the Gli1/Gli2-shRNA group were injected with 200 μl of PBS containing recombinant adenovirus (5 × 10E8 VP/5 μl) and lentivirus (4 × 10E7 VP/50 μl), respectively, through the tail vein after the 14th day of rotavirus fluid injection in mice. The number of mice in each group was 6, and the age of sacrifice was 29 days.

### Quantitative Real-Time PCR Assay

The messenger RNA (mRNA) was isolated from the liver tissue using an Ultrapure RNA Kit (DNaseI; ComWin, Jiangsu, China), and first-strand complementary DNA was synthesized with a HiFiScript complementary DNA Synthesis kit (ComWin, Jiangsu, China). An SYBR Green PCR Master Mix kit (Takara, Liaoning, China) was used for qPCR in a 20-μl reaction volume, and the results were analyzed using a Bio-Rad IQ5 real-time system. β-Actin was used as an internal control, and the relative mRNA expression was quantified by the 2^–ΔΔ*Ct*^ method. The sequences of the qPCR primers are listed in Table 1.

**TABLE 1:** Primer sequence.

Gene		Sequence
Gli1	Forward primer (5′-3′)	5′-CTTTTGCCACCAAGCCAACT-3′
	Reverse primer (5′-3′)	5′-GTCGAGGCTGGCATCAGAAA-3′
Gli2	Forward primer (5′-3′)	5′-AAGACACACCTGCGTTCACA-3′
	Reverse primer (5′-3′)	5′-TCATTGGAGTGAGTGCGGTT-3′
Snail	Forward primer (5′-3′)	5′-ACCTCCAAACCCACTCGGAT-3′
	Reverse primer (5′-3′)	5′-AGACTCTTGGTGCTTGTGGA-3′
Slug	Forward primer (5′-3′)	5′-AGAAGCCCAACTACAGCGAA-3′
	Forward primer (5′-3′)	5′-ATAGGGCTGTATGCTCCCGA-3′
E-cadherin	Reverse primer (5′-3′)	5′-TGTGTGACTGTGAAGGGACG-3′
	Reverse primer (5′-3′)	5′-TGTGTGACTGTGAAGGGACG-3′
Vimentin	Forward primer (5′-3′)	5′-TTCTCTGGCACGTCTTGACC-3′
	Reverse primer (5′-3′)	5′-AGGCTTGGAAACGTCCACAT-3′
α-SMA	Forward primer (5′-3′)	5′-CCACGAAACCACCTATAACAGCA-3′
	Reverse primer (5′-3′)	5′-CTGTGATCTCCTTCTGCATCCT-3′
β-Actin	Forward primer (5′-3′)	5′-GCTACAGCTTCACCACCACA-3′
	Reverse primer (5′-3′)	5′-AAGGAAGGCTGGAAAAGAGC-3′

### Western Blotting

Sclerotic and normal control liver tissues obtained were mechanically minced, homogenized and centrifuged. The protein concentration was detected by the bicinchoninic acid method. After the addition of loading buffer, sodium dodecyl sulfate–polyacrylamide gel electrophoresis was performed, and the proteins were transferred electrophoretically onto polyvinylidene difluoride membranes (Millipore, Bedford, MA, United States). The membranes were subsequently incubated with primary antibodies against GLI1, GLI2, Snail, Slug, α-SMA, vimentin, α-SMA, and E-cadherin overnight at 4°C. The membranes were then washed and incubated with secondary antibody at room temperature (25°C) for an hour. Ultimately, the protein bands were visualized with the enhanced chemiluminescence substrate and exposed to X-ray film.

### Immunofluorescence

The cells on slides were washed and fixed with 4% paraformaldehyde for 15 min, followed by permeabilization with 0.5% Triton X-100 at room temperature. The slides were washed again and blocked with normal goat serum at room temperature and then incubated with primary antibodies (CK19 and α-SMA) followed by secondary antibodies (FITC- and CY3-labeled). After washing again, the stained slides were sealed with anti-fluorescence quenching agent and visualized using a fluorescence microscope (Axio Zoom.V16; Carl Zeiss, Oberkochen, Germany).

### Histology

Samples were taken from the same part of the livers of BA mice, fixed in 10% formalin, embedded in paraffin, and routinely sectioned, followed by hematoxylin and eosin (HE) staining, as well as Masson staining to observe the histological changes and collagen fiber distribution in the liver, respectively. The area, perimeter, mean optical density, integral optical density, and mean grayness of liver collagen fibers were quantified by the Image-Pro Plus 6.0 system (Media Cybernetics, Rockville, MD, United States) according to the criteria for staging pathological liver fibrosis in China (18). The degree of liver fibrosis was assessed by measuring the hydroxyproline content of the samples by the acid hydrolysis method (19).

### Statistical Analysis

The data are presented as the mean ± standard deviation (SD). The Mann–Whitney U test was used for comparisons between two groups with unequal variances. For comparisons of more than two groups, one-way analysis of variance or the Kruskal–Wallis test was performed. Dunnett’s *t*-test was used to examine differences between two selected groups to adjust for bias caused by repeated comparisons. All statistical comparisons were performed using GraphPad Prism software version 8.0 (GraphPad Software 8.0, La Jolla, CA, United States) and SPSS 22.0 (SPSS, Chicago, IL, United States).

## Results

### Altered Expression of Epithelial–Mesenchymal Transition- and Sonic Hedgehog-Related Factors in the Liver Tissue of Biliary Atresia Patients

**FIGURE 1:**
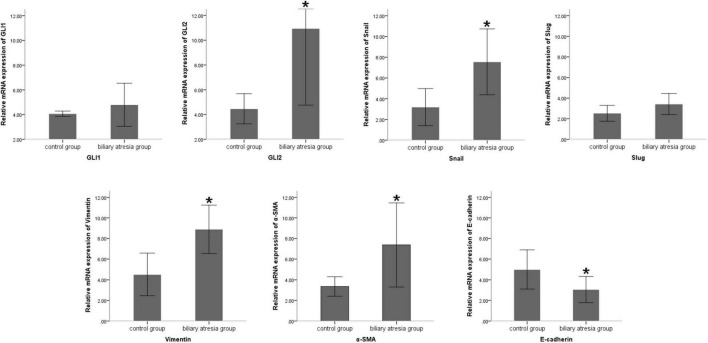
Differences in the mRNA expression of EMT-related factors in liver samples of BA patients and normal controls. Compared to the normal control group, the mRNA expression of Snail, vimentin, and α-SMA was significantly increased and E-cadherin expression was significantly decreased in the liver tissue of BA patients, and there was no significant difference in the expression of GLI1 and Slug. The sample sizes of the biliary atresia group and normal control group were 10 and 5 cases, respectively. The error bars represent SEs of the mean (*P < 0.05).

**FIGURE 2:**
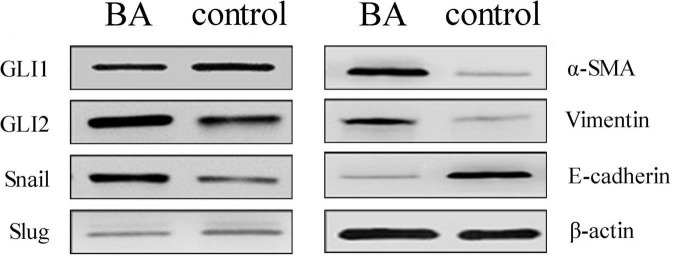
Protein expression changes assessed by western blot in BA patients and normal livers. Compared to the control group, the protein expression of GLI2, Snail, vimentin, and α-SMA was significantly increased, E-cadherin expression was decreased, and there was no significant difference in GLI1 and Slug. The sample size of the BA group was 10 cases and the control group was 5. Representative images of each group are shown in this figure. The molecular weights: GLI1 150 kDa, GLI2 168 kDa, Snail 29 kDa, Slug 30 kDa, α-SMA 42 kDa, vimentin 54 kDa, E-cadherin 98 kDa, and β-actin 42 kDa (BA, biliary atresia).

Compared to the normal control, the mRNA expression of the Shh signaling pathway-related factor GLI2 and EMT-related factors Snail, vimentin, and α-SMA was significantly increased and E-cadherin expression significantly decreased (all *P* < 0.05) in the liver tissue of BA patients, and there was no significant difference in the expression of GLI1 and Slug (Table 2 and Figure 1). The protein expression results showed the same pattern (Figure 2). The results confirmed the presence of EMT in BA and suggested that the Shh signaling pathway was enhanced in BA cirrhosis.

**TABLE 2:** The mRNA expression of EMT-related factors in liver samples of biliary atresia patients and normal controls.

Groups	*N*	Gli1	Gli2	Snail	Slug	Vimentin	α-SMA	E-cadherin
Biliary atresia	10	4.78 ± 1.75	10.96 ± 6.21*^a^*	7.37 ± 4.09*^a^*	3.42 ± 1.03	8.85 ± 2.37*^a^*	7.35 ± 4.09*^a^*	3.00 ± 1.29*^a^*
Normal control	5	4.06 ± 0.28	4.46 ± 1.24	3.34 ± 0.95	2.52 ± 0.76	4.49 ± 2.07	3.38 ± 0.93	4.96 ± 1.91

*N, number.*

*Values are given as the mean ± SD. Compared to the normal control group, ^a^P < 0.05.*

### Altered Expression of Epithelial–Mesenchymal Transition-Related Factors With GLI1/GLI2 Interference in Mouse Intrahepatic Bile Duct Epithelial Cells

**FIGURE 3:**
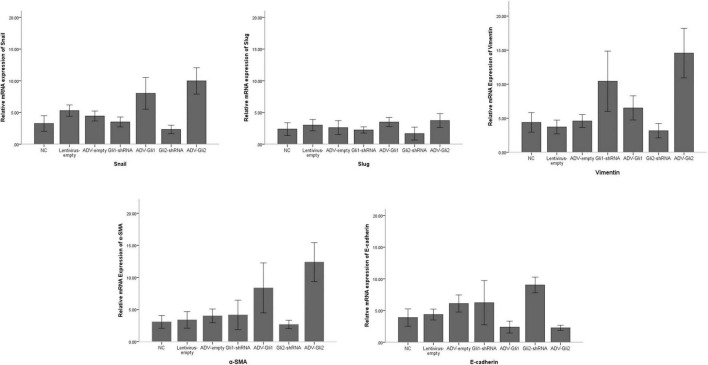
Differences in the mRNA expression of EMT-related factors in mIBECs with GLI1/GLI2 interference. Compared to empty controls, the mRNA expression of Snail, vimentin, and α-SMA significantly increased and that of E-cadherin decreased in mIBECs after GLI2 overexpression (ADV-Gli2); the opposite was observed after GLI2 silencing (Gli2-shRNA), except for α-SMA (all *P* < 0.05). The error bars represent SEs of the mean (NC, normal control).

**FIGURE 4:**
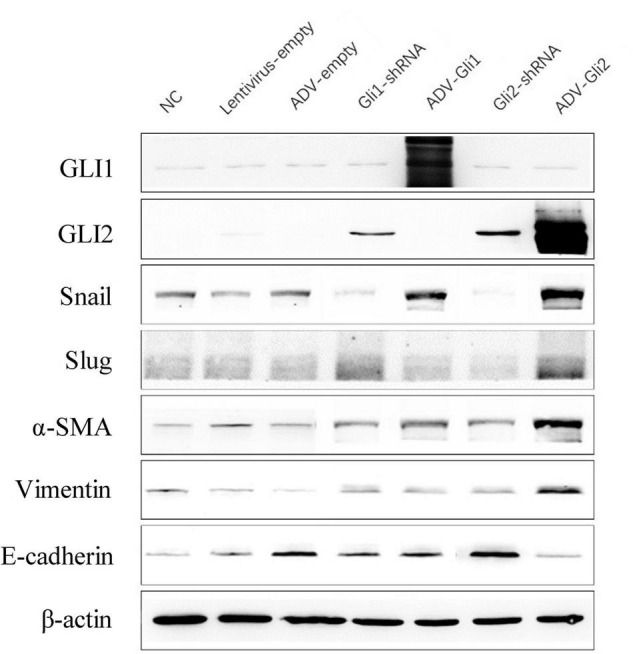
Protein expression changes assessed by western blot after GLI1/GLI2 interference in mIBECs. The protein expression of Snail, vimentin, and α-SMA was almost increased, and E-cadherin expression was decreased after GLI2 overexpression (ADV-Gli2); the opposite was observed after GLI2 silencing (Gli2-shRNA). Representative images of each group are shown in this figure. The molecular weights: GLI1 150 kDa, GLI2 168 kDa, Snail 29 kDa d, Slug 30 kDa, α-SMA 42 kDa, vimentin 54 kDa, E-cadherin 98 kDa, and β-actin 42 kDa (NC, normal control).

The GLI family are key downstream molecules of the Shh signaling pathway; hence, we consider that Shh also promotes BA cirrhosis by altering GLI transcription. Through qPCR analysis, we found that after overexpression of GLI2 expression, the mRNA expression of Snail, vimentin, and α-SMA was significantly increased and that of E-cadherin was reduced compared to controls in mouse intrahepatic bile duct epithelial cells (mIBECs); the opposite was observed after silencing of GLI2, except for α-SMA (all *P* < 0.05). No clear trend of EMT-related factor expression was observed after GLI1 silencing or overexpression (Table 3 and Figure 3). Western blotting showed that the protein presented a similar trend as the mRNA after altering GLI1/2 expression (Figure 4 and Supplementary Figure 2). The above results suggest that the Shh signaling pathway plays an important role in liver fibrosis by regulating the EMT process through GLI2.

**TABLE 3:** The mRNA expression of EMT-related factors in mIBECs with Gli1/Gli2 interference.

Objects	Groups	Snail	Slug	E-cadherin	α-SMA	Vimentin
mIBECs	Normal control	3.21 ± 1.21	2.40 ± 1.00	3.90 ± 1.33	3.12 ± 0.96	4.37 ± 1.46
	Lentivirus-empty	5.21 ± 0.88	3.03 ± 0.87	4.36 ± 0.85	3.46 ± 1.24	3.67 ± 1.04
	ADV-empty	4.32 ± 0.78	2.63 ± 1.10	6.09 ± 1.40	4.09 ± 1.03	4.58 ± 0.95
	Gli1-shRNA	3.42 ± 0.80	2.25 ± 0.48	6.27 ± 3.53	4.19 ± 2.30	10.39 ± 4.42*^a^*
	ADV-Gli1	7.94 ± 2.49*^b^*	3.51 ± 0.73	2.35 ± 1.10*^b^*	8.40 ± 3.87*^b^*	6.47 ± 1.78
	Gli2-shRNA	2.23 ± 0.67*^a^*	1.68 ± 1.03	9.09 ± 1.29*^a^*	2.74 ± 0.61	3.11 ± 1.07*^a^*
	ADV-Gli2	9.89 ± 2.06*^b^*	3.74 ± 1.11	2.30 ± 0.39*^b^*	12.41 ± 3.00*^b^*	14.51 ± 3.63*^b^*

*ADV, adenovirus; mIBECs, mouse intrahepatic bile duct epithelial cells. Values are given as the mean ± SD.*

*Comparison of the GLI1/GLI2-shRNA and lentivirus-empty groups in mIBECs, ^a^P < 0.05; comparison of the ADV-GLI1/GLI2 and ADV-empty groups in mIBECs, ^b^P < 0.05.*

### Altered Messenger RNA Expression of Epithelial–Mesenchymal Transition-Related Factors With GLI1/GLI2 Silencing or Overexpression in Biliary Atresia Mice

**FIGURE 5:**
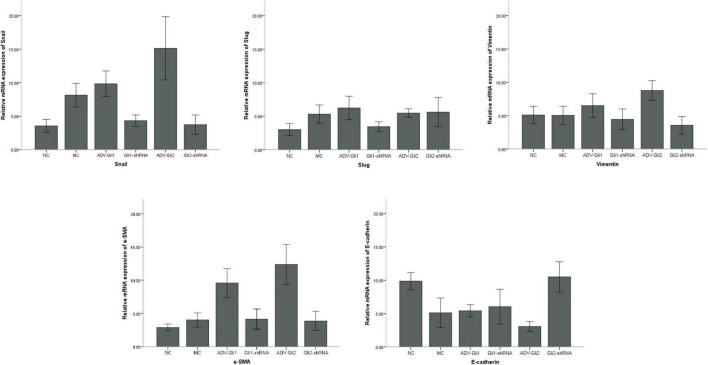
Differences in the mRNA expression of EMT-related factors in mice with GLI1/GLI2 interference. Compared to controls, the mRNA expression of Snail, vimentin, and α-SMA significantly increased and that of E-cadherin decreased in mIBECs after GLI2 overexpression (ADV-Gli2); the opposite was observed after GLI2 silencing (Gli2-shRNA), except for α-SMA (all *P* < 0.05). The error bars represent SEs of the mean (NC, normal control; MC, model control).

**FIGURE 6:**
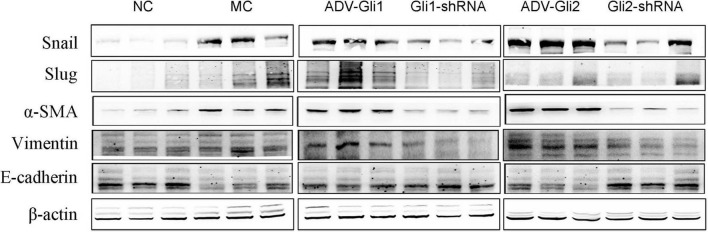
Protein expression changes assessed by western blot after GLI1/GLI2 interference in mice. GLI2 overexpression (ADV-Gli2) was followed by almost increased protein expression of Snail, vimentin, and α-SMA and decreased expression of E-cadherin; the opposite was observed for GLI2 silencing (Gli2-shRNA). The sample size of each group was six cases. Representative images of each group are shown in this figure. The molecular weights: Snail 29 kDa d, Slug 30 kDa, α-SMA 42 kDa, vimentin 54 kDa, E-cadherin 98 kDa, and β-actin 42 kDa (NC: normal control; MC: model control).

In BA mice, silencing or overexpression of GLI2 presented the same trend as in mIBECs, that is, significantly lower Snail and vimentin mRNA expression and significantly higher E-cadherin mRNA expression after overexpression of GLI2 in the livers of BA mice compared with controls; the opposite was observed after silencing of GLI2, except for α-SMA (all *P* < 0.05). Snail and Slug were significantly decreased after GLI silencing, and α-SMA was significantly increased after overexpression, while no significant changes were observed in other EMT-related factors (Table 4 and Figure 5). Western blotting showed that the protein presented a similar trend as the mRNA after altering GLI2 expression. After altering GLI1, the proteins showed similar trends to GLI2 except for Snail and E-cadherin (Figure 6 and Supplementary Figure 3).

**TABLE 4:** The mRNA expression of EMT-related factors in mice with Gli1/Gli2 interference.

Objects	Groups	Snail	Slug	E-cadherin	α-SMA	Vimentin
Mice	Normal control	3.55 ± 0.78	3.00 ± 0.72	9.87 ± 1.04	2.90 ± 0.43	5.11 ± 1.03
	Model control	8.14 ± 1.42	5.32 ± 1.09	5.12 ± 1.78	4.02 ± 0.87	5.05 ± 1.09
	Gli1-shRNA	4.33 ± 0.69*^a^*	3.44 ± 0.59*^a^*	6.04 ± 2.10	4.14 ± 1.23	4.45 ± 1.25
	ADV-Gli1	9.84 ± 1.54	6.24 ± 1.41	5.41 ± 0.74	9.58 ± 1.73*^b^*	6.51 ± 1.43
	Gli2-shRNA	3.73 ± 1.16*^a^*	5.62 ± 1.75	10.51 ± 1.83*^a^*	3.88 ± 1.16	3.56 ± 1.06*^a^*
	ADV-Gli2	15.13 ± 3.81*^b^*	5.48 ± 0.52	3.06 ± 0.59*^b^*	12.40 ± 2.44*^b^*	8.78 ± 1.18*^b^*

*ADV, adenovirus. The sample size of each group was six cases. Values are given as the mean ± SD.*

*Comparison of the GLI1/GLI2-shRNA and model control groups in mice, ^a^P < 0.05; comparison of the ADV-GLI1/GLI2 and model control groups in mice, ^b^P < 0.05.*

### Immunofluorescence of Cytokeratin-19/α–Smooth Muscle Actin in Mouse Intrahepatic Bile Duct Epithelial Cells

**FIGURE 7:**
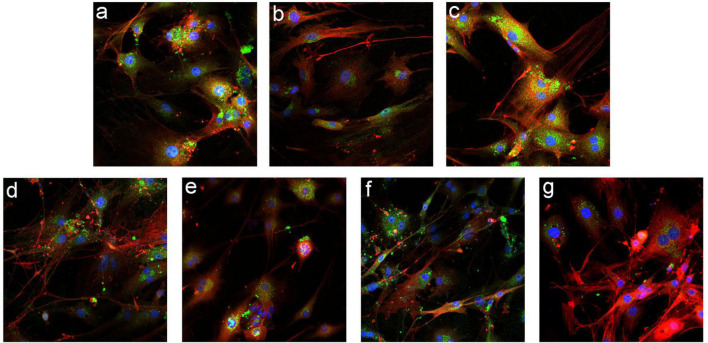
Immunofluorescence staining in mIBECs after overexpression and silencing of GLI1 and GLI2 (FITC- and CY3-labeled, magnification: ×100). **(a)** Normal control. **(b)** The empty lentivirus. **(c)** ADV-empty. **(d)** Gli1-shRNA. **(e)** ADV-Gli1. **(f)** Gli2-shRNA: compared to **(b)**, the fluorescence intensity of CK19 (green) was increased and that of α-SMA (orange) was decreased. **(g)** ADV-Gli2: compared to **(c)**, the fluorescence intensity of α-SMA was significantly increased and that of CK19 was significantly decreased.

Immunofluorescence detection (Figure 7) showed that among the mIBEC groups, the fluorescence intensity of the epithelial cell marker CK19 (green) was increased and that of the myofibroblast marker α-SMA (orange) was decreased in the Gli2-shRNA group compared with the lentivirus control, suggesting that more bile duct epithelial cells and fewer mesenchymal cells existed after GLI2 silencing. In contrast, the fluorescence intensity of α-SMA was significantly increased and that of CK19 was significantly decreased in the ADV-Gli2 group compared with the adenovirus control, suggesting a decrease in bile duct epithelial cells, which may be converted to mesenchymal cells through EMT.

### Histopathological Changes in the Liver in All Groups of Mice

**FIGURE 8:**
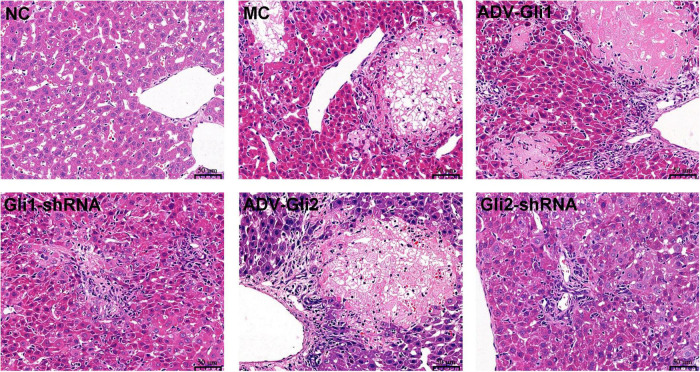
Hematoxylin and eosin (HE) staining of liver tissues of mice in different groups (magnification: ×400). Normal control (NC): clear structure and no obvious fibrous hyperplasia; model control (MC): variable clusters of necrotic lesions in the liver lobules and mild fibrosis in the portal area; ADV-Gli1: slight fibrosis in the portal area accompanied by inflammatory cell infiltration; Gli1-shRNA: slight fibroplasia and inflammatory cell infiltration in the portal area; ADV-Gli2: some hepatocytes were necrotic and formed necrotic nodules surrounded by fibrous cysts, and the structure of the portal area was disordered; Gli2-shRNA: slight fibrous hyperplasia around the portal area. Representative images of each group are shown in this figure.

Hematoxylin and eosin staining of the mice liver (Figure 8) shows that the structure of the normal control group was clear and no obvious fibrous hyperplasia was observed in the ductal area; in the BA model control group, multiple clusters of necrotic foci were seen in the liver lobules, with mild fibrosis and inflammatory cell clusters in the portal area; in the ADV-Gli1 and Gli1-shRNA groups, mild fibroplasia and inflammatory cell infiltration were observed in the portal area; in the ADV-Gli2 group, the structure of the portal area was blurred and disorganized, the canals were narrowed, and some necrotic hepatocytes formed nodules surrounded by fibrous encapsulation; in the Gli2-shRNA group, some of the canals in the portal area were occluded with blurred demarcation of the liver lobules and mild fibrous hyperplasia around the portal area.

**FIGURE 9:**
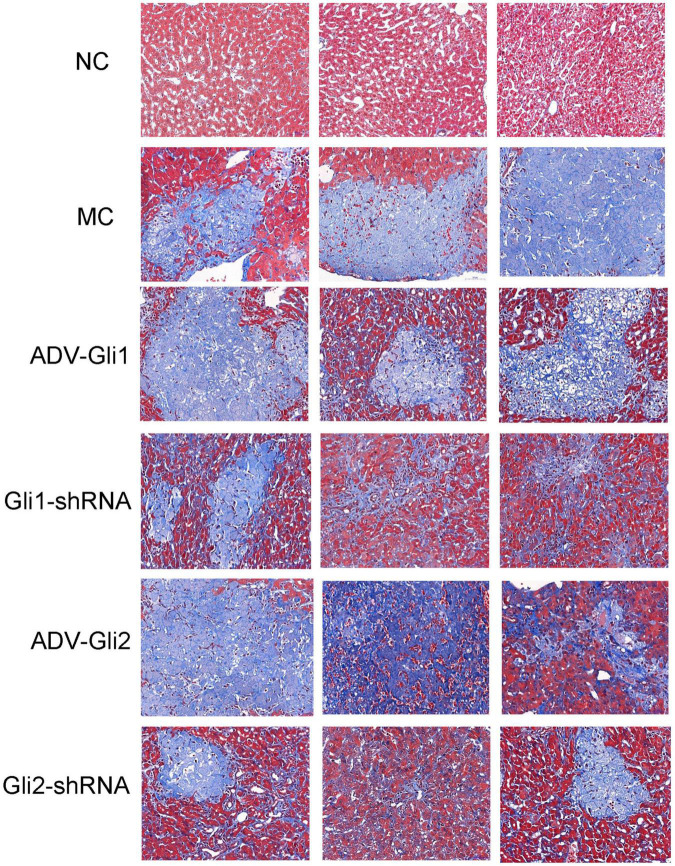
Masson staining of liver tissues of mice in different groups (magnification: ×400). The percentages of liver fibrous tissue expression area in the ADV-Gli2 group > ADV-Gli1 group > model control (MC) group > Gli1-shRNA group > Gli2-shRNA group > normal control (NC) group. Among them, the results of the ADV-Gli2 group and Gli2-shRNA group were significantly different compared with the model control (*P* < 0.05).

**FIGURE 10:**
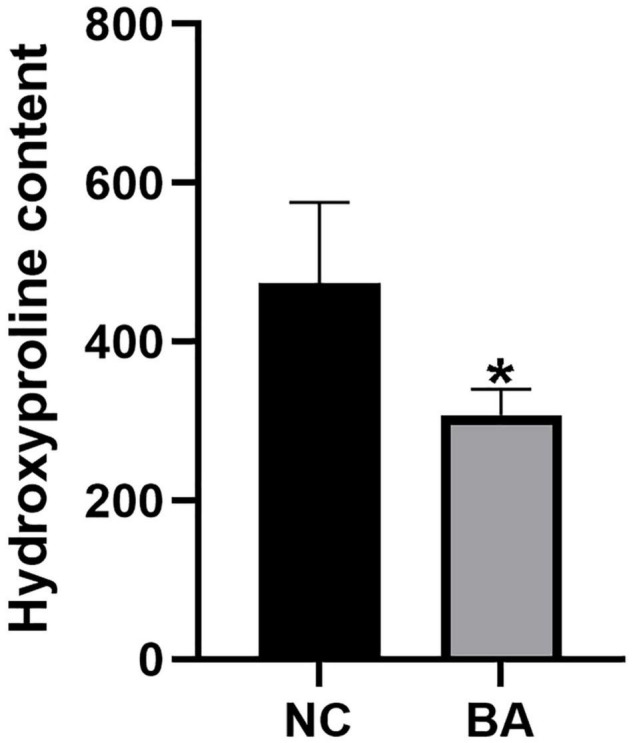
Hydroxyproline quantification in the livers of mice by the acid hydrolysis method. The hydroxyproline levels were significantly lower in the BA (*n* = 10) group than in the NC (*n* = 3) group. *P < 0.05.

After Masson staining of the mice liver tissue, the optical density and area of the acquired images were measured by the Image-Pro Plus 6.0 system, and the percentages of liver fibrous tissue expression area were calculated (Table 5 and Figure 9), with the trend as follows: ADV-Gli2 group > ADV-Gli1 group > BA model group > Gli1-shRNA group > Gli2-shRNA group > normal control group. The results were statistically significant in the ADV-Gli2 group (*P* = 0.03) and Gli2-shRNA group (*P* = 0.04) compared with the model group, while those in the ADV-Gli1-overexpressing group (*P* = 0.27) and Gli1-shRNA group (*P* = 0.21) compared with the model group were not statistically significant. The hydroxyproline content in the control (*n* = 3) and experimental groups (*n* = 10) was 474.50 ± 101.12 and 307.33 ± 32.78, respectively (*P* < 0.01; Figure 10).

**TABLE 5:** Quantification of fibers and mucus as a ratio to whole area at the liver in each group of mice.

Groups	Area percentage
NC	1.03 ± 0.58
MC	33.02 ± 11.39
ADV-Gli1	39.81 ± 5.67*^a^*
Gli1-shRNA	26.06 ± 1.29*^b^*
ADV-Gli2	49.81 ± 8.57*^c^*
Gli2-shRNA	17.55 ± 0.66*^d^*

*NC, normal control; MC, model control. Values are given as the mean ± SD.*

*^a^ADV-Gli1 vs. MC, t = −1.20, P = 0.27; ^b^Gli1-shRNA vs. MC, t = 1.36, P = 0.21; ^c^ADV-Gli2 vs. MC, t = −2.63, P = 0.03; ^d^Gli2-siRNA vs. MC, t = 3.03, P = 0.04.*

The above results suggest that the enhancement of GLI2 expression promotes the liver fibrosis process in BA.

## Discussion

Although the etiology of BA is currently unclear, most studies now agree that BA is a fibrotic disease that causes atresia of the major extrahepatic and intrahepatic biliary ducts and leads to cirrhosis (13). Liver fibrosis is a damage healing process that occurs when the liver is chronically injured, forming pseudolobule and nodules that gradually evolve into cirrhosis and eventually liver failure (20). Previous studies suggested that mesenchymal cells play an important role in liver fibrosis, and activation and regeneration of myofibroblasts are the central steps in chronic liver fibrogenesis with BA (2, 12). Liver fibrogenesis in BA is a complex interactive process in which EMT and Shh signaling pathways may play an important role, but more details remain to be elucidated.

Epithelial–mesenchymal transition is considered to be one of the important sources of mesenchymal cells and a well-recognized mechanism of myofibroblast formation in injured tissues (21). Evidence has shown that EMT is also involved in liver fibrogenesis in BA (8, 22, 23). First described by Greenberg and Hay in 1982, EMT refers to the morphological transformation of epithelial cells into fibroblasts or mesenchymal cell phenotypes, along with the acquisition of the ability to migrate (24). EMT plays important roles in embryonic development, histogenesis, and various pathological processes (25). In the physiological state, EMT is an important wound-healing mechanism; however, abnormal persistence of EMT is pathological and can cause fibrosis and sclerosis in tissues and organs (26). With the finding of evidence of EMT in rodent livers, BECs have rapidly become the focus of EMT studies (27, 28). Although the EMT process in fibrogenesis with BA has been studied extensively, the etiology and molecular mechanisms are still not well established. However, evidence suggests that the activation of the Shh signaling pathway may be important in this process.

The Hedgehog family is a group of highly conserved genes that encode proteins that have been identified as essential morphogenes for embryonic development and tissue remodeling in adult tissue (29). The Shh gene is a member of the Hedgehog gene family, and evidence has shown that Shh signaling is activated in damaged livers and that its expression parallels the degree of fibrosis (30). In particular, Shh ligand expression is significantly upregulated in BECs during cholestatic cirrhosis (27, 31). Cui et al. (32) found significantly lower expression of the *GPC1* gene in BECs from children with BA than in normal controls and determined that glypican 1, a heparan sulfate proteoglycan encoded by the *GPC1* gene, could regulate Shh signaling, suggesting that the Shh signaling pathway may be important in liver fibrogenesis in BA.

The Shh signaling pathway consists mainly of the transmembrane protein receptor complex composed of the SHH, Ptch, and SMO proteins and the downstream transcription factor GLI family (33). Three GLI protein homologs exist in mammals, of which GLI1 and GLI2 mainly perform activating functions that GLI3 mainly inhibits (34). Aberrant activation of the Shh signaling pathway contributes to abnormal expression of target genes by increasing the transcriptional levels of GLI1 and GLI2, ultimately leading to the development of disease (35). Snail plays an important role in cell survival, immune regulation, and stem cell biology, but the best known function is the induction of the EMT process through the inhibition of E-cadherin transcription (15). The GLI family was found to rapidly induce the transcription of Snail in mouse epidermal cells, while Snail also maintains the expression of GLI through a positive feedback loop (36–38). Because of the close association of the Shh signaling pathway and EMT process, we hypothesized that EMT and Shh mutually regulate the process of BA cirrhosis.

In this study, first, by comparing the mRNA and protein quantification of EMT and Shh-related factors in the livers of patients with BA and normal controls, the results confirmed that the mRNA and protein expression of GLI2, Snail, vimentin, and α-SMA were almost all higher in the BA group, while E-cadherin was significantly lower, suggesting the existence of EMT and the enhancement of the Shh signaling pathway in BA cirrhosis. Subsequently, altered expression of EMT-related factors was observed in the same trend as in the livers of patients with BA after overexpression of GLI1 and GLI2 by adenovirus in mIBECs and BA mice, with more significant effects of the GLI2 gene. The results suggest that the Shh signaling pathway can regulate the EMT process in mIBECs and the livers of BA mice through its downstream factors GLI1 and GLI2, and the enhanced expression of GLI2 could be a key event in the amplification of this process. Follow-up immunofluorescence results of mIBECs and histological analysis of livers of BA mice also demonstrated increased fibrosis markers in mIBECs and more pronounced liver fibrosis in BA mice after overexpression of GLI2.

In contrast, when GLI2 was silenced with lentivirus, the EMT-related factors and liver fibrosis markers in mIBECs changed in the opposite trend to those after overexpression, and the degree of liver fibrosis was partially reversed in BA mice. The results again illustrated the important role of GLI2 in regulating the EMT process in livers with BA *via* the Shh signaling pathway and suggested that the EMT process could be reversible to a certain extent. Taken together, these findings suggest that the process of liver fibrosis in BA can be reversed by reversing EMT and converting cells from a mesenchymal phenotype to an epithelial phenotype, that is, mesenchymal epithelial transformation (MET).

However, there are some limitations in our study. First, the number of liver biopsies from BA patients included in this study was only 10, and the smaller number may cause data bias. Second, although we suggest that liver fibrosis in BA may be reversible, the hypothesis is based on current observational results and has not been confirmed in *in vivo* models, so further experiments are also needed to explore and confirm our hypothesis. Nevertheless, our findings provide a new target and basis for the prevention and treatment of liver fibrosis in BA in the future and hopefully will help improve the prognosis of children with BA.

## Data Availability Statement

The original contributions presented in the study are included in the article/Supplementary Material, further inquiries can be directed to the corresponding author.

## Ethics Statement

The studies involving human participants were reviewed and approved by the Institutional Review Board and Ethical Committee at the West China Hospital of Sichuan University in China. Written informed consent to participate in this study was provided by the participants’ legal guardian/next of kin.

## Author Contributions

JS contributed to conceptualization, supervision, and writing—review and editing. JS, WJ, and WQ contributed to the methodology. PS, WJ, and WQ performed the experiments. PS, WJ, and ZY analyzed the data. PS contributed to writing—original draft. All authors contributed to the article and approved the submitted version.

## Conflict of Interest

The authors declare that the research was conducted in the absence of any commercial or financial relationships that could be construed as a potential conflict of interest.

## Publisher’s Note

All claims expressed in this article are solely those of the authors and do not necessarily represent those of their affiliated organizations, or those of the publisher, the editors and the reviewers. Any product that may be evaluated in this article, or claim that may be made by its manufacturer, is not guaranteed or endorsed by the publisher.

## Supplementary Material

Supplementary Figure 1 Purity identification of isolated BECs by immunofluorescence with CK19. CK19 showed red fluorescence, and cell purity >90% was acceptable.

Supplementary Figure 2 The grayscale ratio of EMT-related factors in mIBECs with Gli1/Gli2 interference. Compared to empty controls, the band intensities of Snail, vimentin, and α-SMA significantly increased and that of E-cadherin decreased in mIBECs after GLI2 overexpression (ADV-Gli2); the opposite was observed after GLI2 silencing (Gli2-shRNA), except for vimentin (all *P* < 0.05). The error bars represent SEs of the mean (NC, normal control).

Supplementary Figure 3 The grayscale ratio of EMT-related factors in mice with Gli1/Gli2 interference. Compared to the model control (MC), the band intensities of Snail, vimentin, and α-SMA significantly decreased and that of E-cadherin increased after GLI2 silencing (Gli2-shRNA); the opposite was observed after GLI2 overexpression (ADV-Gli2), except for E-cadherin (all *P* < 0.05). The error bars represent SEs of the mean (NC, normal control).

## Abbreviations

Shh: Sonic hedgehog
EMT: epithelial–mesenchymal transition
BA: biliary atresia
ADV: adenoviruses
mRNA: messenger RNA
mIBECs: mouse intrahepatic bile duct epithelial cells
CK19: cytokeratin-19
α-SMA: α–smooth muscle actin
HE: hematoxylin and eosin.
