# Supplementary figures and images for: The Role of GLI in the Regulation of Hepatic Epithelial–Mesenchymal Transition in Biliary Atresia
Source: Front Pediatr. 2022 May 26;10:861826. doi: 10.3389/fped.2022.861826 (PMC9178093; doi:10.3389/fped.2022.861826)

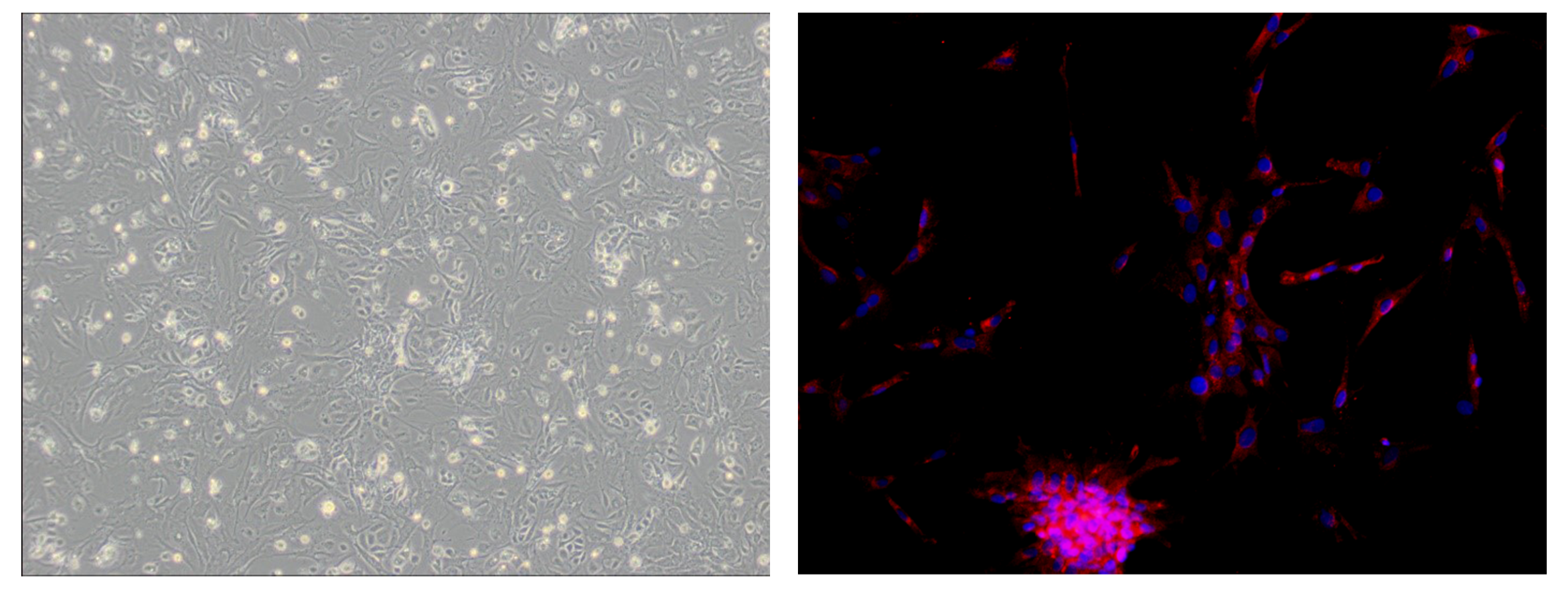

Supplement: Supplementary Figure 1 — Purity identification of isolated BECs by immunofluorescence with CK19. CK19 showed red fluorescence, and cell purity >90% was acceptable. [file Image_1.TIF]

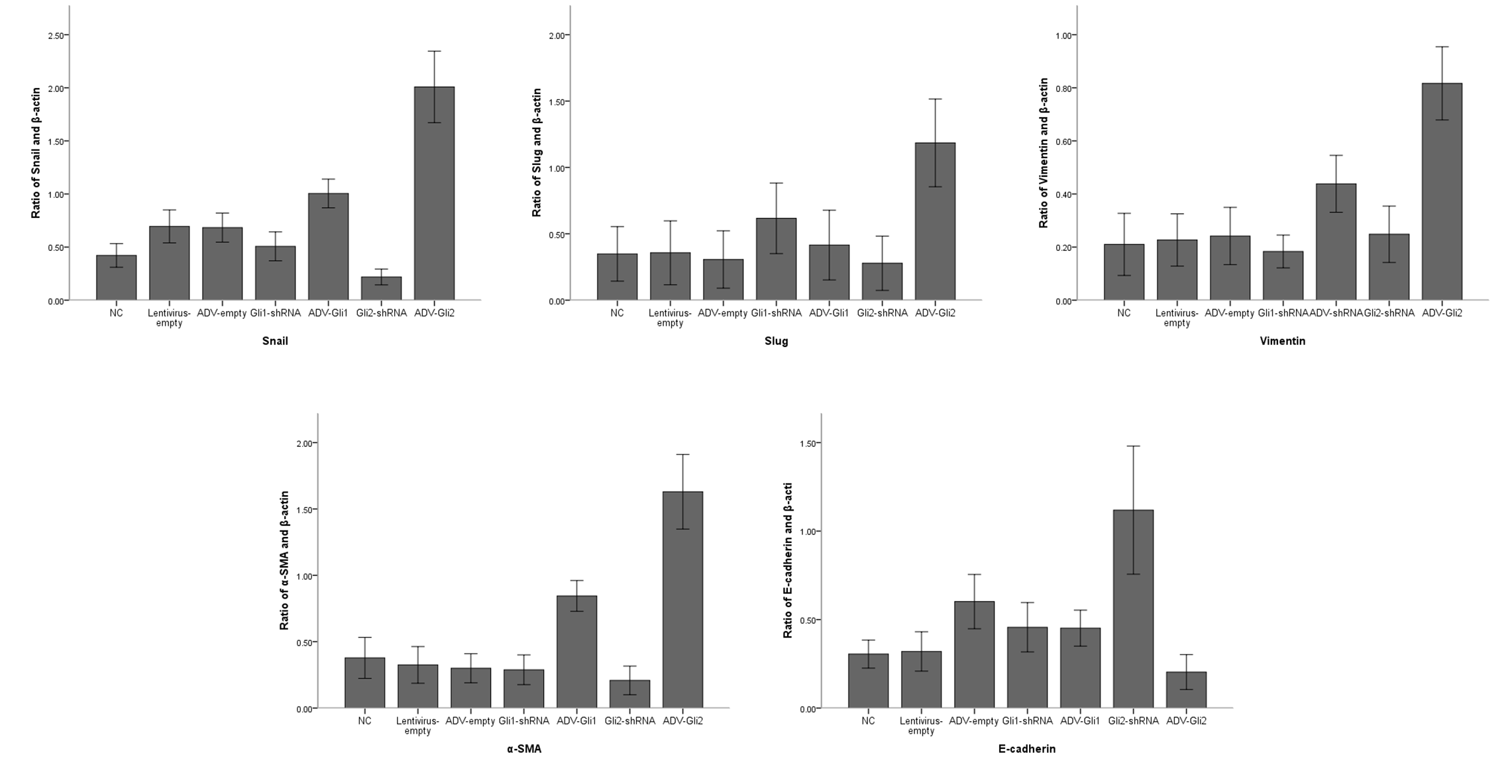

Supplement: Supplementary Figure 2 — The grayscale ratio of EMT-related factors in mIBECs with Gli1/Gli2 interference. Compared to empty controls, the band intensities of Snail, vimentin, and α-SMA significantly increased and that of E-cadherin decreased in mIBECs after GLI2 overexpression (ADV-Gli2); the opposite was observed after GLI2 silencing (Gli2-shRNA), except for vimentin (all P < 0.05). The error bars represent SEs of the mean (NC, normal control). [file Image_2.TIF]

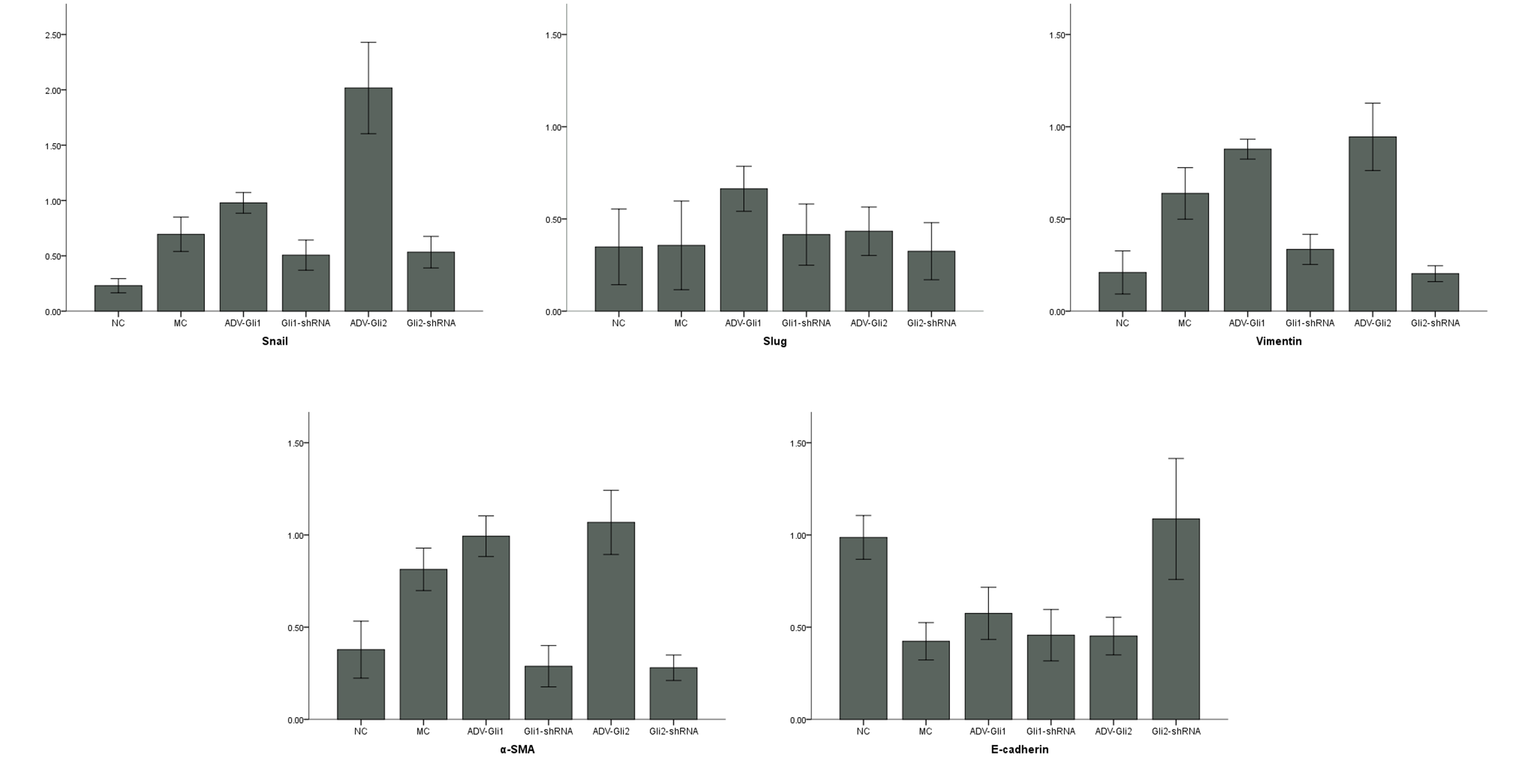

Supplement: Supplementary Figure 3 — The grayscale ratio of EMT-related factors in mice with Gli1/Gli2 interference. Compared to the model control (MC), the band intensities of Snail, vimentin, and α-SMA significantly decreased and that of E-cadherin increased after GLI2 silencing (Gli2-shRNA); the opposite was observed after GLI2 overexpression (ADV-Gli2), except for E-cadherin (all P < 0.05). The error bars represent SEs of the mean (NC, normal control). [file Image_3.TIF]
